# Aortoduodenal Syndrome: A Ruptured Abdominal Aortic Aneurysm Causing a Small Bowel Obstruction With Stroke-Like Symptoms

**DOI:** 10.7759/cureus.117631

**Published:** 2026-10-08

**Authors:** Jacqueline Sippel, Ashley Foreman, Christopher S Kiefer, James Scheidler

**Affiliations:** 1 Emergency Medicine, West Virginia University School of Medicine, Morgantown, USA; 2 Emergency Medicine, Summa Health System, Akron, USA

**Keywords:** abdominal aortic aneurysm, aortoduodenal syndrome, case report, ct angiography, small bowel obstruction, stroke-like symptoms

## Abstract

Aortoduodenal syndrome is a rare complication of abdominal aortic aneurysm (AAA) in which extrinsic compression of the duodenum results in gastric outlet or small bowel obstruction. We present the case of a 73-year-old man who presented to the emergency department with dizziness, profuse vomiting, cerebellar neurologic findings, and absent palpable lower-extremity pulses. Imaging demonstrated a large infrarenal AAA with findings concerning for contained rupture and extrinsic compression of the duodenum, resulting in gastric and duodenal dilation and at least a partial small bowel obstruction. The patient underwent emergent endovascular aortic repair with stent graft placement and was successfully discharged home on postoperative day 1. This case highlights the variable presentation of aortoduodenal syndrome, which may include gastrointestinal obstructive symptoms, hemodynamic instability, vascular compromise, and stroke-like exam findings. Given the rarity of this condition, clinicians should maintain a high index of suspicion for aortic pathology when these findings occur concurrently. Although computed tomography angiography is generally preferred when feasible, the choice of imaging modality should account for patient stability, availability, and diagnostic accuracy. Prompt recognition and definitive vascular intervention are essential to prevent catastrophic complications associated with ruptured AAA.

## Introduction

Aortoduodenal syndrome is an exceedingly rare and serious clinical entity characterized by a mechanical upper gastrointestinal obstruction caused by the direct extrinsic compression of the duodenum by a large abdominal aortic aneurysm (AAA) [1]. First documented by Canadian physician William Osler in 1905, fewer than 50 cases of this syndrome have been reported in medical literature [1-3]. Patients present with a combination of symptoms that range from vomiting and inability to pass flatus, as seen with bowel obstructions, to, as our case illustrates, hemodynamic instability, acute ischemic findings in the lower extremities, and varying neurological symptoms, raising suspicion for aortic pathology [1]. Swift diagnosis is imperative given the potentially fatal outcome of aortic rupture. Aortoduodenal syndrome can be diagnosed with multiple different imaging modalities, the efficiency of which can be debated based on availability and patient stability. Patients with this diagnosis should be managed with vascular intervention. In addition to stabilizing a precarious aortic aneurysm, this intervention can decompress the bowel obstruction, even without resolution of the aneurysm caliber [2].

We report a patient with a contained ruptured AAA presenting as aortoduodenal syndrome accompanied by stroke-like neurologic symptoms. To our knowledge, this represents only the fourth reported case of a ruptured AAA causing this syndrome [3] and the first describing associated neurologic deficits, based on our literature search, which did not identify previously published cases describing a similar presentation.

## Case presentation

A 73-year-old man presented to the emergency department complaining of two days of dizziness with standing and one day of profuse non-bloody, non-bilious emesis. Initial vital signs upon arrival were notable for a non-invasive blood pressure of 82/60 mmHg and a heart rate of 116 beats/min. However, repeat vitals shortly thereafter normalized: blood pressure of 121/97 mmHg with a heart rate of 98 beats/min. No intervention was required at that time to correct hemodynamics. On physical examination, he was noted to have a soft, non-distended abdomen with only mild right upper quadrant tenderness with no palpable masses or signs of peritonitis. He also demonstrated right-sided dysdiadochokinesia without nystagmus and mildly impaired left-sided heel-to-shin coordination. Bilateral lower extremity peripheral pulses could not be palpated, but bilateral posterior tibial and anterior tibial Doppler signals were obtained via a handheld Doppler device. No dorsalis pedis Doppler signals were able to be identified.

Given the above neurological symptoms and vomiting, there was concern for possible carotid or vertebral artery dissection causing vertebrobasilar insufficiency. Computed tomography angiography (CTA) of the head and neck was ordered with a computed tomography of the abdomen and pelvis (CT AP) without intravenous contrast to further delineate mild nonspecific abdominal pain with associated history of recurrent emesis. The CT AP was ordered without contrast and performed in the delayed post-contrast phase after the CTA as the contrast from the CTA was expected to "run off" into the abdominal images, thereby providing some distal vascular opacification. Given the prominent neurological symptoms with concern for vertebrobasilar insufficiency, CTA of the head and neck was prioritized over CT AP, as there was lower concern for primary intra-abdominal pathology. This reasoning was also why abdominal CTA or bedside aortic ultrasound was not performed at that time. The patient's laboratory workup was notable for leukocytosis, a drop in hemoglobin and hematocrit, as well as an increase in blood urea nitrogen and creatinine, values displayed in Table 1.

**Table 1:** Laboratory values on the day of presentation compared to baseline values from nine days prior

Lab test	Normal values	Day of presentation	Baseline (9 days prior)
White blood cells	4.5-11.0×10^3^/uL	14.2×10^3^/uL	-
Hemoglobin	14-18 g/dL	13.2 g/dL	14.9 g/dL
Hematocrit	42-50%	37.6%	42.2%
Blood urea nitrogen	8-23 mg/dL	29 mg/dL	16 mg/dL
Creatinine	0.6-1.2 mg/dL	1.36 mg/dL	1.1 mg/dL

Imaging results were notable for the following: for CTA intracranial, mild intracranial atherosclerotic disease without high-grade stenosis, aneurysm, or vascular malformation; for CTA extracranial, approximately 50% stenosis at the origin of the right vertebral artery, atherosclerotic disease with ulcerative plaques within the aortic arch, and mild bilateral carotid bifurcation atherosclerotic disease; and for CT AP without contrast (Figures 1-2), large infrarenal AAA measuring approximately 9.1×9.1×12.6 cm with extension into the aortic bifurcation with concern for contained leak/rupture with a hyperdense crescent sign consistent with an intramural hematoma and diffuse stranding around the infrarenal AAA. The stomach and duodenum were dilated with abrupt narrowing as the duodenum crossed the large AAA consistent with at least partial small bowel obstruction (SBO).

**Figure 1:**
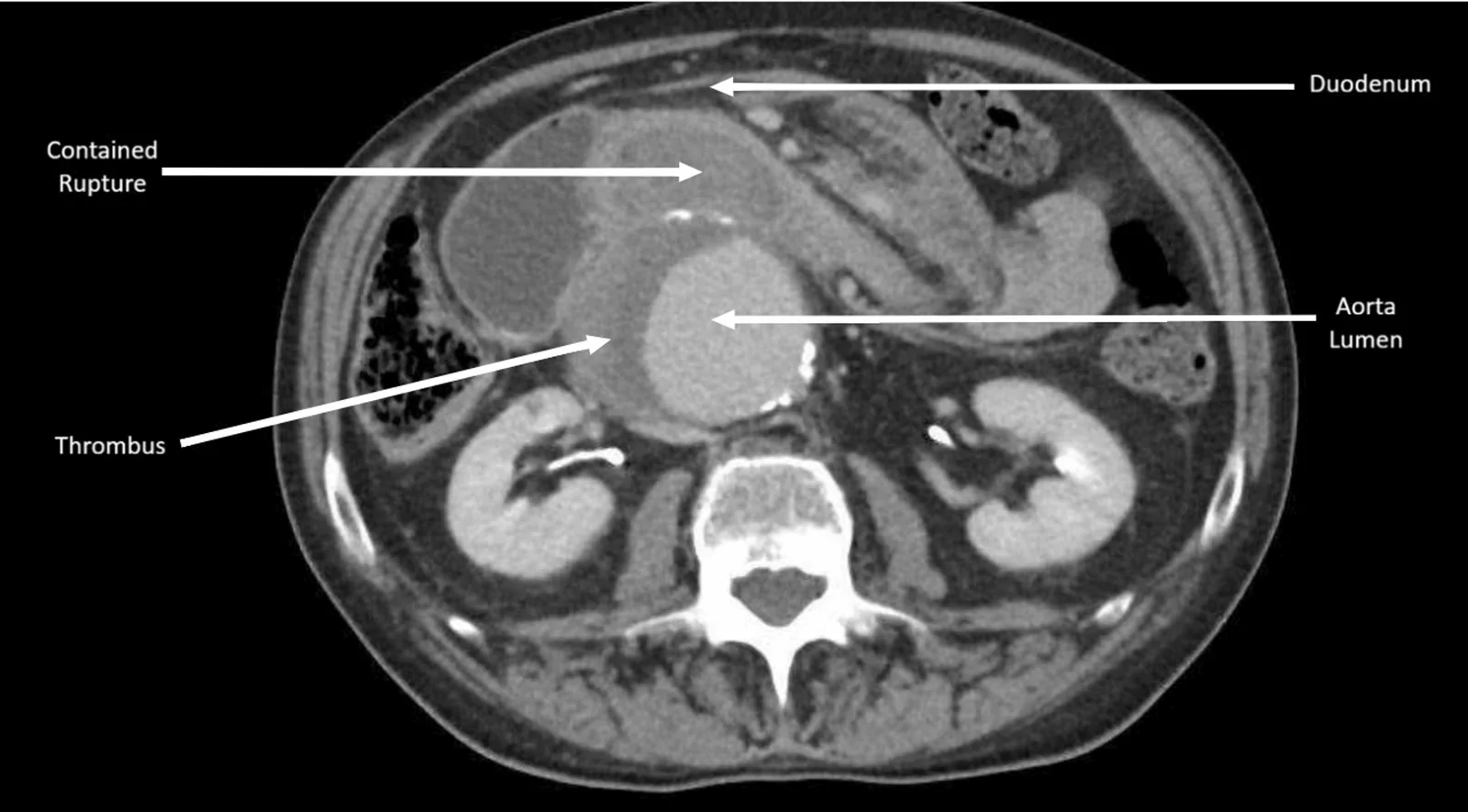
Computed tomography axial cut showing the contained ruptured abdominal aortic aneurysm and the extrinsic compression of the duodenum

**Figure 2:**
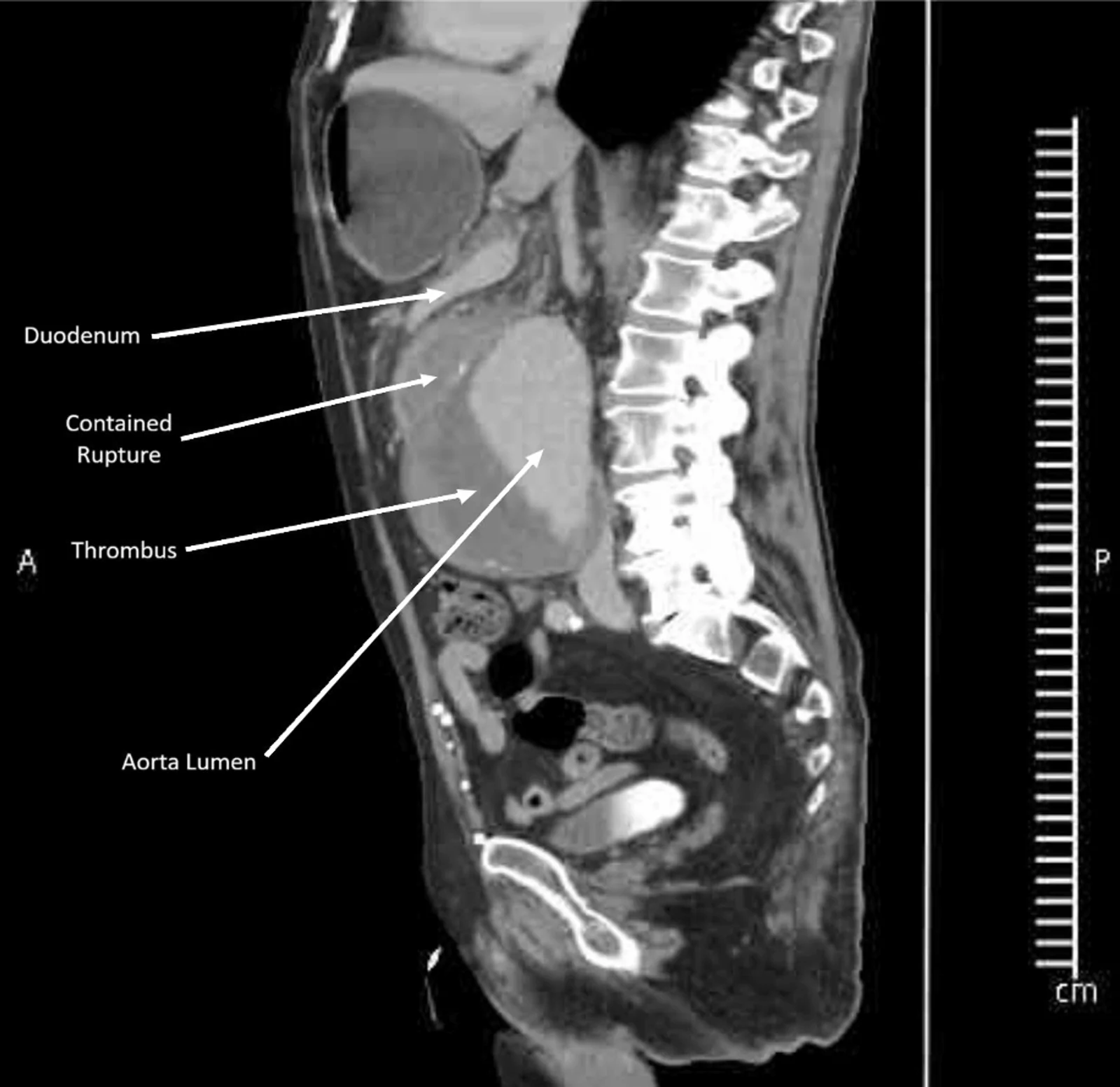
Computed tomography sagittal cut showing the abdominal aortic aneurysm with contained rupture and mural thrombus causing compression of the duodenum

Once the AAA with a contained rupture was identified, Vascular Surgery was emergently consulted, and the patient was started on a titratable esmolol drip at 50 mcg/kg/min until the desired hemodynamic response was achieved. Upon further questioning, the patient denied any previous history or diagnosis of AAA. He was taken directly from the emergency department to the endovascular surgery suite, where he underwent angiography that confirmed a ruptured AAA. Subsequently, he underwent endovascular aneurysm repair (EVAR) with placement of a 16×20×11 mm Gore Excluder stent graft (Newark, Delaware, United States) and a 29×3 mm extender stent. No nasogastric tube was placed, and the patient did not require any blood products. The patient tolerated the procedure well and, ultimately, was discharged home on postoperative day 1 after he was able to tolerate oral intake without further emesis.

## Discussion

In 1905, a rare entity known as aortoduodenal syndrome was first described by Dr. William Osler who reported the case of an elderly woman who was found to have an "enormously dilated stomach due to pressure of an aneurysm on the duodenum" [1]. The case we present of a ruptured AAA causing aortoduodenal syndrome is only the fourth reported case to our knowledge [3] and the first to describe associated stroke-like symptoms. The exact mechanism of obstruction remains uncertain, but several hypotheses have been proposed in the literature. One proposed mechanism involves the direct mechanical compression of the duodenum, which is pushed against the abdominal wall or superior mesenteric artery as the underlying aneurysm enlarges [4,5]. Another hypothesis is that the posterior wall of the third section of the duodenum (D3) becomes adherent to the lateral wall of the aneurysm leading to compression of the duodenal lumen as the aneurysm expands [6]. We postulate that the relatively rapid progression of the patient's gastric outlet obstruction symptoms was related to expansion of the aneurysm. The preceding neurologic symptoms raised concern for vertebrobasilar insufficiency in the setting of hypotension that we suspect was caused by the contained rupture; however, this proposed relationship remains inferential, and a definitive causal association between the ruptured aneurysm and neurologic findings cannot be established.

The current suggested workup for aortoduodenal syndrome begins with a CT using intravenous contrast for initial evaluation, with strong consideration of CTA. Due to the rarity of aortoduodenal syndrome, it is difficult to say definitively which imaging modality would be the best for diagnosis. Therefore, when evaluating the utility of different imaging modalities, we looked at the sensitivity and specificity for both SBO and AAA separately, focusing specifically on the use of CT imaging and ultrasound. However, these diagnostic accuracy data are derived from studies evaluating SBO and AAA independently and may not directly reflect the diagnostic performance of these modalities in patients with aortoduodenal syndrome. For the purpose of this case study, magnetic resonance imaging (MRI) will not be discussed as it is not the preferred imaging modality for AAA in general due to the overall cost, time constraints, and variable availability at medical care facilities.

Starting with the evaluation for SBO, CT had a sensitivity of 91% and a specificity of 89% [7]. Ultrasound had a sensitivity of 92% and a specificity of 96%; however, ultrasound does have limitations to its use due to operators' ability to obtain and interpret images [8,9].

When looking at imaging modalities for AAA, we evaluated non-contrast CT in addition to CTA and ultrasound. Non-contrast CT has a sensitivity of 82-88% and a specificity of 97-98% for identifying a non-ruptured AAA, and it has a sensitivity of 85% and a specificity of 81% for a ruptured AAA [10,11]. CTA has a nearly 100% sensitivity and specificity for identifying a non-ruptured AAA with a sensitivity of 91.4% and a specificity of 93.6% for a ruptured AAA [10,12]. Ultrasound is used for the identification of a non-ruptured AAA, though its use is limited due to patient body habitus and the presence of bowel gas [13]. It has a sensitivity ranging from 57% to 70% and a specificity of 99% [10]. Point-of-care ultrasound can be used to identify a ruptured AAA with a sensitivity of 97.8% and a specificity of 97%; however, it has a low sensitivity in detecting a retroperitoneal hemorrhage which occurs in 70-90% of ruptured AAA [12-14].

Interventions for aortoduodenal syndrome have changed over time, and published case reports suggest that EVAR may be a potential treatment option in select patients as symptom resolution can occur despite the absence of aneurysmal shrinkage [2], as demonstrated in our patient.

## Conclusions

Aortoduodenal syndrome is an exceedingly rare physiologic phenomenon. The presentation of this condition can vary from the classic symptoms of an SBO to neurologic sequelae or even a mixture of both as was present in our patient. Due to the rarity of this condition, there is no universally agreed-upon imaging modality; however, the ideal case would be to obtain a CTA as it has a near 100% sensitivity and specificity for unruptured AAA and greater than 91% sensitivity and 93% specificity for ruptured AAA. We acknowledge the utility of point-of-care ultrasound in recognizing this condition, especially in patients who are unstable and would be otherwise unsafe to place in a CT scanner. However, there are limitations to its use including the operator's ability to obtain necessary views (whether that is due to operator skill, patient body habitus, or even overlying bowel gas causing obscuration of the field of view) and to interpret the images obtained. Finally, treatment approaches for this condition have changed over time, and limited case-based evidence suggests that EVAR alone may be considered in select patients, as demonstrated by the favorable clinical outcome in our case.

## References

[1] Osler W (1905). Aneurysm of the abdominal aorta. Lancet.

[2] Kawaguchi Y, Oba Y, Suzuki Y, Tamaki M, Koyama Y, Kitamura H (2020). Successful endovascular repair for abdominal aortic aneurysm presenting as aortoduodenal syndrome. Ann Vasc Dis.

[3] Franga DL, Wiginton JG 4th (2016). A rare presentation of ruptured abdominal aortic aneurysm leading to aortoduodenal syndrome. J Vasc Surg Cases.

[4] Parashar K, Gandhi D, Nepal P, Sapire J, Ahuja K, Siddiqui I (2020). Abdominal aortic aneurysm and its association with duodenal obstruction: aortoduodenal syndrome. BJR Case Rep.

[5] Taylor SG, van Rij AM, Woodfield JC (2016). Duodenal obstruction associated with an abdominal aortic aneurysm. J Vasc Surg Cases Innov Tech.

[6] Cahill K, Roche-Nagle G, MacEneaney P, McGreal G (2009). Upper gastrointestinal obstruction secondary to aortoduodenal syndrome owing to a noninflammatory abdominal aortic aneurysm. Vascular.

[7] Li Z, Zhang L, Liu X, Yuan F, Song B (2019). Diagnostic utility of CT for small bowel obstruction: systematic review and meta-analysis. PLoS One.

[8] Gottlieb M, Peksa GD, Pandurangadu AV, Nakitende D, Takhar S, Seethala RR (2018). Utilization of ultrasound for the evaluation of small bowel obstruction: a systematic review and meta-analysis. Am J Emerg Med.

[9] Long B, Gottlieb M (2021). Accuracy of ultrasonography for the diagnosis of small bowel obstruction. Am Fam Physician.

[10] Liisberg M, Diederichsen AC, Lindholt JS (2017). Abdominal ultrasound-scanning versus non-contrast computed tomography as screening method for abdominal aortic aneurysm - a validation study from the randomized DANCAVAS study. BMC Med Imaging.

[11] Adam DJ, Bradbury AW, Stuart WP, Woodburn KR, Murie JA, Jenkins AM, Ruckley CV (1998). The value of computed tomography in the assessment of suspected ruptured abdominal aortic aneurysm. J Vasc Surg.

[12] Fernando SM, Tran A, Cheng W (2022). Accuracy of presenting symptoms, physical examination, and imaging for diagnosis of ruptured abdominal aortic aneurysm: systematic review and meta-analysis. Acad Emerg Med.

[13] Perae GO, Corneli M (2026). Non-invasive techniques in diagnosis and treatment of patients with abdominal aortic aneurysm. e-J. Cardiol. Pract..

[14] Gibbons RC, Singh G, Donuru A, Young M (2026). Abdominal aortic aneurysm imaging. StatPearls [Internet].

